# Dynamic mechanical thermal analysis (DMTA) of the hybrid epoxy/carbon-fibers nanocomposites for satellite structures

**DOI:** 10.1038/s41598-026-47147-9

**Published:** 2026-04-17

**Authors:** Mohammed Gamil, W. M. Farouk, Ahmed Abu-Oqail, Mohamed Abu-Okail, Ghaith Al-Hawajreh, Waleed El-Sallamy, Abou Bakr Elshalakany

**Affiliations:** 1 Mechanical Engineering Department, Faculty of Engineering-Shoubra, Benha University, Cairo 11614, Egypt; 2 Mechanical Engineering Department, Faculty of Engineering-Benha, Benha University, Benha, Egypt; 3 Mechanical Production Department, Faculty of Technology and Education, Beni-Suef University, Beni-Suef, Egypt; 4 Korean Egyptian Faculty of Industry and Energy Technology, Beni-Suef Technological University, Beni-Suef, 62521 Egypt; 5 Mechatronics Technology Department, Higher Technological Institute Beni-Suef, Beni-Suef, Egypt; 6 Mechanical and Industrial Engineering Department, Faculty of Engineering and Technology, Applied Science Private University, Amman, 11937 Jordan; 7 Production Engineering and Printing Technology Department, Akhbar El Yom Academy, Giza, 12451 Egypt; 8 Faculty of Energy and Industrial Technology, October Technological University, Giza, Egypt

**Keywords:** Carbon fibers, Epoxy, Nanocomposites, Hand layup, DMTA, Aerospace applications, Engineering, Materials science, Nanoscience and technology

## Abstract

The current paper investigates the influence of the reinforced nanocomposites, including TiO_2_, ZrO_2_, SiO_2_, and graphite, on dynamic mechanical and thermal behaviour of epoxy/carbon fiber nanocomposites fabricated by hand layup technique. To investigate the effect of the reinforced nanocomposites on the DMTA, several weight percentages (1.5 wt%, 3 wt%) were taken into consideration. The microstructural features of fabricated specimens were examined through an optical microscope. High-resolution SEM imaging, elemental mapping analysis, and fractography were conducted to analyze fiber distribution, interfacial bonding, and failure mechanisms. Superior damping factor and a higher transition temperature were observed for epoxy/carbon-reinforced fiber with 3 wt% TiO_2_. In comparison to the 1.5 wt% ZrO_2_ nanocomposite, the 3 wt% ZrO_2_ nanocomposite shows improved mechanical properties overall, including lower tensile compliance, a slightly higher T_g_, and a greater complex viscosity. This implies that better stiffness and thermal stability are the outcomes of increasing ZrO_2_ concentration. Adding 3 wt% SiO_2_ enhances the stiffness of material’s and stability of thermal up to T_g_. Generally, an elevate in reinforcement content (from 1.5 wt% to 3 wt%) results in a higher damping factor. At higher temperatures over 80 °C nearly, the tensile compliance improved and increased by the addition of 3 wt% SiO_2_, 1.5 wt% graphite, 1.5 wt% TiO_2_, 3 wt% TiO_2_, 3 wt% ZrO_2_, and 1.5 wt% ZrO_2_, consequently. ZrO_2_ (3 wt%) shows better results compared to TiO_2_ and SiO_2_, highlighting its effectiveness at higher concentrations. The optical and SEM micrographs show minimal agglomeration and good nanoparticle distribution, confirming successful composite preparation.

**Supplementary Information:**

The online version contains supplementary material available at 10.1038/s41598-026-47147-9.

## Introduction

In the past decades, hybrid fiber-reinforced polymers (HFRPs) with nano-additives have become an effective alternative for conventional metallic components used in satellite structures^1–4^. Across various reinforcement fibers, synthetic fibers Offer significant potential in satellite applications because of their lightweight nature, high-strength, superior-stiffness and specific modulus, as well as excellent wear and heat resistance^5^. Carbon fibers are widely utilized as reinforcing agents in thermosetting polymers, especially epoxy, to enhance its dynamic mechanical thermal properties^6–8^. Furthermore, the addition of nano-powder such as TiO_2_, ZrO_2_, SiO_2_, and graphite to the fabricated fiber-reinforced polymers (FRPs) have a great attraction in the past years due to their ability to enhance the dynamic mechanical thermal properties^9–11^.

Rankin et al.^12^ investigate that the optimal performance for the produced multifunctional carbon fiber composites reinforced with 1.0 wt% TiO_2_ has enhanced damping and strength with significant improvements in interlaminar shear strength, highlighting the potential of this scalable method for satellite applications. In addition, Al-Zubaydi et al.^13^ explained in their study that TiO_2_ plays a vital role in enhancing the resistance of wear and impact strength of carbon fiber reinforced epoxy. 4 wt% TiO_2_ shows the best performance. However, excessive addition increased liquid uptake, indicating the need for optimized nanoparticle content to achieve balanced properties for wear-resistant applications, especially satellite applications.

Wu et al.^14^ stated that ZrO_2_ coating at 1.0 wt% significantly enhanced the carbon fiber/epoxy composite, achieving uniform particle dispersion and increasing interfacial shear strength by 41.3% and fracture toughness by 257.6%, confirming improved interphase strength and toughness.

Silicon oxide (silica) nanoparticles have incorporated great interest in the last few years because of their high specific surface area, low toxicity, stable surface chemistry, and ease of manufacturing processes. It is currently used in many industrial applications as reinforced nanoparticles, such as aerospace applications. For example, Qin et al.^15^ elucidated that the uniform deposition of SiO_2_ as reinforced nanoparticles on the carbon fibers surfaces improves the interlaminar shear strength of carbon/epoxy composites to a great extent, up to 22%, when compared to the control specimen.

Gkikas et al.^16^ fabricate a polymer matrix with a dispersion of carbon nanotubes to improve the thermo-mechanical characteristics. A remarkable increase in the storage modulus and glass transition temperature is achieved. Hidayah et al.^17^ incorporated carbon nanotubes inside laminated composites of glass/kenaf/epoxy and carbon/kenaf/epoxy. The storage modulus was achieved ~ 42% in the glassy region for ink/MWCNT/GKKG and a shift in glass transition temperature from 85.7 to 90.1 °C for ink/MWCNT/CKKC was also observed. Moreover, Cai et al.^18^ impregnate the epoxy resin into porous graphene nanoplatelet paper to develop a new graphene nanocomposite. In comparison to plain epoxy, the resultant graphene nanocomposite exhibited a 170% increase in storage modulus. Bafakeeh et al.^19^ Integrated alumina and graphene nanoplatelets into epoxy resin to fabricate high-performance, multifunctional nanocomposites for satellite applications. Typically, the addition of these nano-additives increases the hardness and tensile strength to a great extent. For instance, the tensile strength was elevated by 97 MPa due to the addition of 3 wt% alumina.

Recent studies have systematically explored the effect of nanofillers on the mechanical and thermomechanical performance of FRPs. For example, Fuseini et al.^20^ reviewed epoxy nanocomposite coatings with multiple hybrid nanofillers and emphasized the importance of filler dispersion and surface chemistry on mechanical and tribological performance, highlighting.

Comprehensive reviews on carbon nanotube–reinforced composites have outlined synthesis, dispersion strategies, and performance enhancements achievable with CNTs, corroborating the significance of filler type and morphology on mechanical properties^21^.

Further, the role of different nanomaterials (oxides, silica, carbon-based fillers) on mechanical, thermal, and interfacial characteristics has been highlighted in recent literature, indicating that tailored nanofiller selection can improve stiffness, damping, and high-temperature stability in polymer matrices^22^.

Recent advancements in polymer–fiber nanocomposites further support the trends showed in the present study. Several recent works have demonstrated that the incorporation of nanoscale oxides, carbons, and hybrid fillers can significantly affect the thermo-mechanical, morphological, and functional performance of epoxy-based and fiber-reinforced systems. These studies collectively emphasize the critical role of nanoparticle morphology, surface chemistry, and dispersion state in controlling modulus enhancement, thermal stability, damping behavior, and interfacial bonding within composite structures. Additionally, the literature highlights the growing interest in optimizing nanofiller types, loadings, and processing techniques to balance stiffness, toughness, and high-temperature performance for advanced engineering applications. The findings of these investigations corroborate the present results, confirming that carefully selected nanofillers, when properly dispersed, can provide notable improvements in mechanical integrity, thermal response, and failure behavior. The referenced works also demonstrate the increasing relevance of nanocomposites in aerospace panels, structural brackets, electronic housings, and multifunctional components, thereby underscoring the broader applicability and technological importance of the composite systems examined in this study^23–30^.

The epoxy/carbon-fiber nanocomposites investigated in this study are particularly relevant for lightweight structural and semi-structural components used in satellite systems. These include primary and secondary panel skins, equipment support brackets, instrument housings, antenna substrates, vibration-damping frames, and thermally stable enclosures. Such components require a combination of high stiffness, thermal resistance, and controlled damping under fluctuating orbital and launch environments. The enhancement in thermomechanical performance observed in this work, especially improvements in T_g_, storage modulus, and high-temperature damping, directly supports the suitability of these nanomodified composites for these satellite applications.

A comparative study of various nanoparticles is necessary for better understanding of their influence on the thermomechanical properties of the composite material made of carbon fibers and epoxy resin. Although many researchers have worked on the thermomechanical properties of individual nanoparticles, comparative studies of various oxide-based and carbon-based nanoparticles under the same conditions of composite formation and testing are limited^31–43^. Studies of TiO_2_, ZrO_2_, SiO_2_, and graphite nanoparticles within the same composite matrix would reveal the influence of the chemical composition of the nanoparticles, their morphology, and their interaction with the matrix on the thermomechanical properties of the matrix-fiber combination. DMTA is one of the techniques that could provide an overall understanding of the influence of the nanoparticles on the thermomechanical properties of the composite material. Complex modulus (E*), tensile compliance (D*) Damping factor (*Tan δ*), and complex viscosity (η*) could provide overall information about the thermomechanical properties of the composite material. Each parameter reflects a different aspect of the viscoelastic response such as stiffness, energy dissipation, deformation ability, and molecular mobility. Considering all parameters together provides a more comprehensive evaluation of thermomechanical performance, which is necessary for designing the composite material for the construction of satellite structures.

The goal of the current work is to provide a novel hybrid epoxy/carbon fiber nanocomposite reinforced with one of the following nanocomposites: TiO_2_, ZrO_2_, SiO_2_, and graphite by hand layup technique to enhance the DMTA. Two different weight fractions (1.5 wt% to 3 wt%) from the nanoparticles (TiO_2_, ZrO_2_) were considered for the fabricated nanocomposite, while 3 wt% SiO_2_ and 1.5 wt% graphite were added individually to form another two new nanocomposites. The fabricated samples are studied from the point of view of the DMTA in *Tan δ*, E*, D*, and η*. Surface structural of the fabricated samples was also evaluated through an optical microscope and SEM. In addition, the fracture surfaces were also analyzed.

This study provides a novel comparative evaluation of four different nanoparticle types (TiO_2_, ZrO_2_, SiO_2_, graphite) incorporated into carbon-fiber/epoxy laminates. While previous work has typically focused on single-filler systems or modified fibers, this research simultaneously investigates multiple viscoelastic parameters (E, D*, Tan δ, η*) under identical conditions. The results offer a unique selection framework for optimizing nanofiller choice for thermomechanical stability in aerospace structures, thereby addressing a gap in literature.

## Materials and methods

Epoxy (Kemapoxy Cast – CMB Company, Cairo, Egypt) was utilized as the matrix, while carbon fiber (Arab World for Financial Investments Company, Cairo, Egypt) functioned as the strengthening material. Furthermore, four different nano-powders including TiO_2_, ZrO_2_, SiO_2_, and graphite (Nano Gate Company for Nano Materials, Cairo, Egypt) were incorporated individually as nano-additives to enhance the DMTA to launch, vibrational, impact, and thermal loads.

The selection of oxide and carbon-based nanoparticles follows demonstrated trends in recent composite research. Reviews and experimental investigations have shown that carbon nanotubes and other nanofillers such as graphene or oxide particles can significantly influence modulus, damping, and thermal behavior in epoxy matrices, often between ~ 0.5–3 wt% loadings, beyond which agglomeration effects dominate^22^. These nanoparticle types were selected based on their mechanical and thermal stability, availability in reproducible nano-scale forms, and compatibility with epoxy matrices. TiO_2_ and ZrO_2_ contribute high stiffness and thermal resistance, SiO_2_ improves interphase bonding, and graphite enhances damping and crack-deflection mechanisms. Their proven relevance in structural composites makes them suitable candidates for aerospace applications.

The physical and mechanical characteristics of carbon fibers and additives nano-powders are illustrated in Table 1. The TiO_2_ and ZrO_2_ nano-powders have spherical shape with diameter size 43.3 nm, and 50 nm, respectively. In addition, their densities were 4.10 gm/cm^3^, 5.68 gm/cm^3^. Moreover, they had respective purities of around 98% and 97%. SiO_2_ and graphite nano-powders were flake shape with diameter size 45 nm, and 95 nm, respectively. The density of SiO_2_ and graphite were 2.00 gm/cm^3^, and ~ 2.2 gm/cm^3^, respectively. The purity SiO_2_ and graphite nano-powders were about ~ 98% and ~ 96%, respectively. These values are consistent with those commonly reported in the literature for aerospace-grade epoxy systems and high-strength carbon-fiber reinforcements^37,44,45^.

**Table 1:** Properties of nano-powders, epoxy and carbon fibres.

Properties	TiO_2_	ZrO_2_	SiO_2_	Graphite	Epoxy	Carbon fiber
Density (g/cm^3^)	4.5	5.68	2.00	2.2	1.16	1.6
Melting point (°C)	1580	2823	1720	3700	-	-
Average particle size (nm)	43.3	50	45	95	-	-
Tensile strength (MPa)	333.3	711	320	280	110	2400
Young modulus (GPa)	230	250	180	1 × 10^6^	4.1	72.3
Purity (%)	98	97	98	96		
Appearance	White	White	White	Dark gray	White	Black

Prior to mixing, the morphology of each nanoparticle type was examined. TiO_2_ and ZrO_2_ particles displayed spherical morphology with narrow size distributions. SiO_2_ and graphite exhibited flake-like structures consistent with their known physical forms. These observations confirmed manufacturer specifications and ensured no unexpected particle elongation or aspect-ratio variations.

All the fabricated specimens have 60% carbon fibers with 40% epoxy-based matrix, engineered into 30-layered structures. TiO_2_, ZrO_2_, SiO_2_, or graphite nanoparticles at concentrations of 1.5 wt% and 3 wt% were incorporated into the epoxy matrix, resulting in modified compositions with 38.5% or 37% epoxy, respectively, while maintaining the carbon fiber content at 60%. The details of these innovative composites are presented in Table 2.

A 30-layer laminate configuration was selected to ensure sufficient structural rigidity, load-carrying capacity, and thermal stability for typical satellite panel and bracket applications. Multi-ply carbon-fiber/epoxy laminates within the range of 24–32 layers are commonly reported for aerospace secondary structures, as this thickness provides the required balance between flexural stiffness, impact tolerance, and weight efficiency while maintaining dimensional stability under thermo-mechanical loading^2,37,39,46–49^. Previous studies have shown that laminates in this thickness range exhibit improved vibration resistance and reduced micro-buckling sensitivity, making them suitable for components exposed to launch loads and orbital thermal cycling. Therefore, a 30-layer configuration was chosen to match standard aerospace composite design practices and to ensure meaningful mechanical and thermal evaluation under conditions representative of satellite structures.

The selected nanoparticle contents of 1.5 wt% and 3 wt% were chosen based on established trends in nanocomposite processing and performance optimization. Numerous studies report that nanofiller additions below ~ 1 wt% often produce minimal improvements due to insufficient particle–matrix interfacial area, while loadings above 3 wt% typically result in agglomeration, increased viscosity, poor wet-out, and degraded mechanical properties^38,50,51^. Experimental findings in oxide- and carbon-based nanofilled epoxy systems consistently show that the 1–3 wt% range provides the most effective balance between dispersion quality, interphase formation, and mechanical enhancement without significantly impairing processability. Preliminary trials performed in this work confirmed that concentrations above 3 wt% led to visible particle clustering and higher resin viscosity, making uniform laminate fabrication difficult. Therefore, 1.5 wt% and 3 wt% were selected as practical and literature-supported reinforcement levels to evaluate the effect of controlled nanoparticle loading on the thermomechanical response of the carbon-fiber/epoxy composites.

The composition range was selected based on preliminary dispersion testing. SiO_2_ at 1.5 wt% produced negligible improvements compared to the neat composite, so only 3 wt% was used. Graphite at 3 wt% led to notable agglomeration during sonication, reducing laminate quality. Therefore, 1.5 wt% was selected as the highest fully dispersible and mechanically stable concentration.

**Table 2:** Weight fractions of composite contents.

Condition	TiO_2_	ZrO_2_	SiO_2_	Graphite	Epoxy	Carbon fiber	Total
a	-	-	-	-	40%	60%	100%
b	1.5%	-	-	-	38.5%	60%	100%
c	3%	-	-	-	37%	60%	100%
d	-	1.5%	-	-	38.5%	60%	100%
e	-	3%	-	-	37%	60%	100%
f	-	-	3%	-	37%	60%	100%
g	-	-	-	1.5%	38.5%	60%	100%

**Fig. 1:**
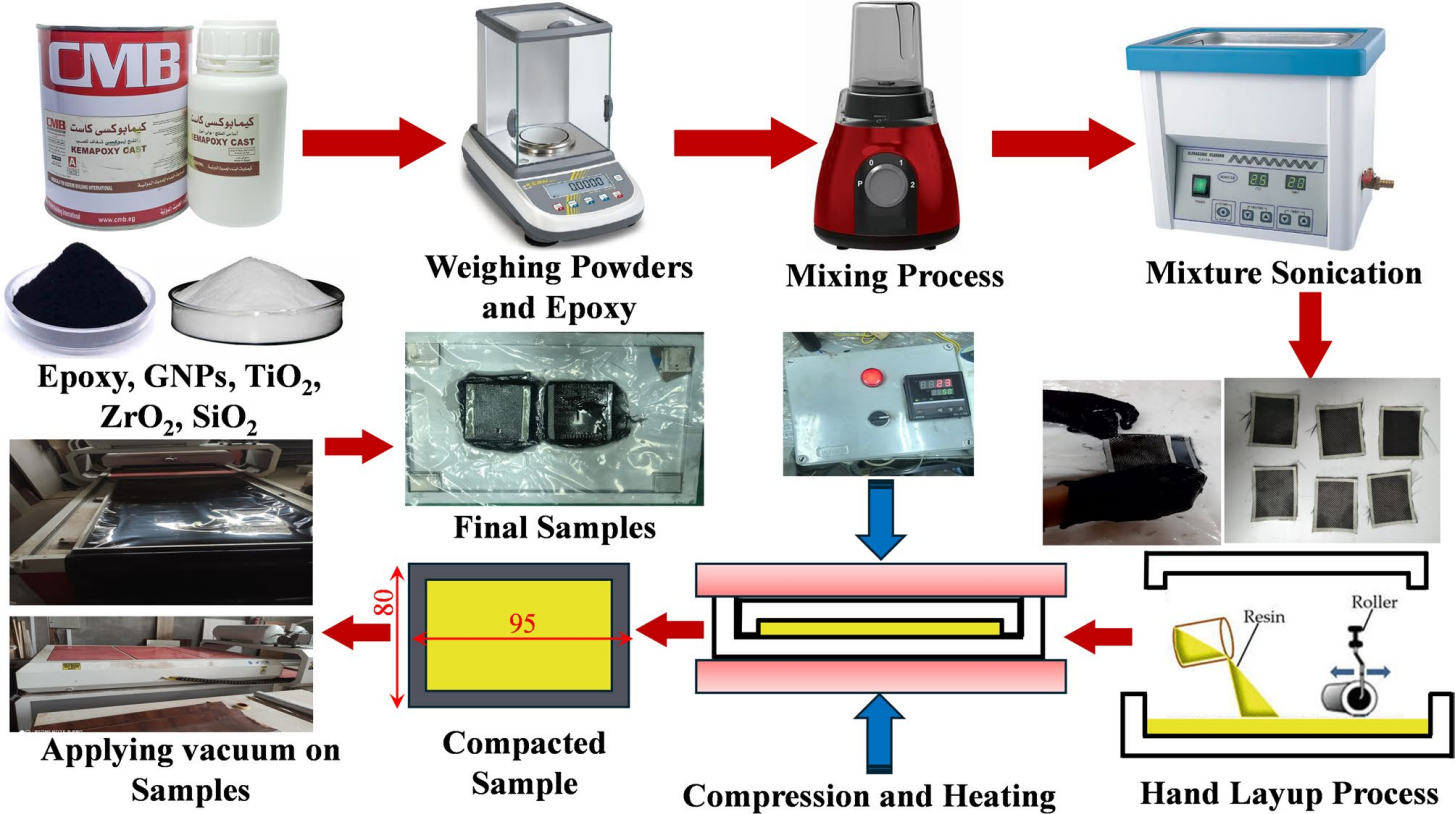
Sequential schematic illustration for the fabrication process.

To enhance the bond with epoxy, the nanoparticles undergo a pre-treatment using a non-reactive modifier, a blend of stearic acid and an ethyl acetate solution. A mechanical mixer blends stearic acid with an ethyl acetate solution for 30 min, then integrates the resultant mixture with nanoparticles for an additional 30 min, as depicted in Fig. 1. Next, the nanoparticles are rinsed with an ethyl acetate solution to effectively eliminate the stearic acid. To ensure a uniform scattering of nano-powders within the epoxy matrix, the blend is subjected to an intensive ultrasonication process. A Henan Lanphan (UP200S) ultrasonic stirrer was employed at 500 RPM for 30 min to obtain a consistently uniform dispersion of the nano-additives. In addition, the sonication process was adjusted to 0.5 cycle/sec with a 70% amplitude. Moreover, sonication was carried out for 3 h in order to prevent the nanoparticle agglomeration. A sonication duration of 3 h was selected to ensure adequate deagglomeration and homogeneous dispersion of the nanoparticles within the epoxy matrix. Sonication times in the range of 1–4 h are commonly employed in epoxy–nanoparticle systems to break apart nanoparticle clusters and promote stable suspensions, especially for oxide and carbonaceous fillers with high surface energy^52,53^. Previous studies have shown that shorter sonication periods (< 1 h) often result in incomplete dispersion, whereas excessively long durations (> 4 h) may lead to resin overheating, polymer chain scission, or nanoparticle surface modification, all of which can negatively affect composite performance. The 3-hour duration used in this study falls within the optimal range reported in the literature and was verified through preliminary trials to produce stable dispersions without signs of resin degradation. Therefore, this duration was selected as a practical and evidence-based choice to achieve effective nanoparticle distribution prior to laminate fabrication.

The epoxy/nanoparticles were precooled in an ice bath before the sonication process to prevent the epoxy from degrading. After that, the hardener was mixed with the epoxy resin in a 1:2 weight ratio. Finally, the blend underwent a curing process at 55 °C for 15 min, ensuring optimal crosslinking and stability. The epoxy/carbon fiber nanocomposite structures were carefully fabricated using the hand lay-up technique, ensuring precise layering and uniform distribution of materials. The nanocomposite structures were built up layer by layer, precisely stacked until the desired thickness and strength were attained.

The technique of vacuum bagging is utilized on epoxy/carbon fiber nanocomposites to reduce voids and air bubbles, provide uniform pressure distribution, enhance nanoparticle distribution, improve fiber-to-resin ratio, and improve dimensional accuracy and surface quality. We then apply a controlled curing cycle to ensure optimal material properties and structural integrity. After the curing process, test samples are precisely cut from the fabricated epoxy/carbon fiber nanocomposite with the desired dimension for each test. The samples were examined by optical microscopy (OM), scanning electron microscopy (SEM), energy-dispersive X-ray spectroscopy (EDS), and elemental mapping analysis both before and after DMTA testing to assess their structural integrity and composition.

A 3-point bending test with a dual cantilever setup machine (DMA/SDTA861e) was used to conduct the DMTA tests on the fabricated samples to assess their viscoelastic properties to ensure their suitability for satellite applications. The DMTA samples were precisely cut to 50 mm × 10 mm × 2.3 mm according to the ASTM D4065 standard for accurate and reliable evaluation. Before testing, the fabricated samples were stabilized at an isothermal temperature for 5 min at 1 Hz to achieve thermal equilibrium and exact readings. The DMTA test was then performed by gradually increasing the temperature from 30 °C to 150 °C and the dwell time from 5 to 575 min. A heating rate of 4 °C/min and a nitrogen flow rate of 20 ml/min were conducted to ensure precise thermal characterization. A computer-based data acquisition system was employed to precisely control machine operations and seamlessly record experimental data in real time. Three samples per case were analyzed to ensure accuracy, and the average values were determined for reliable assessment.

## Results and discussion

### Dynamic mechanical thermal analysis (DMTA)

**Fig. 2:**
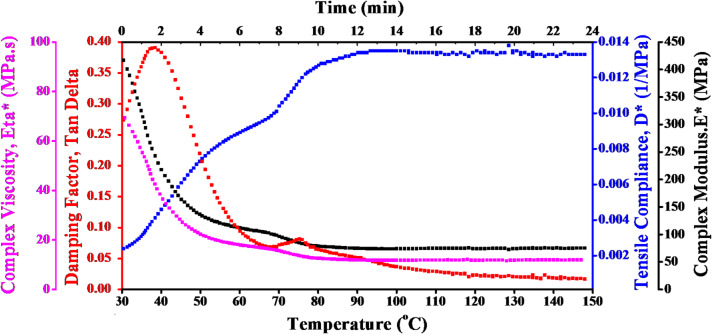
DMTA of epoxy/carbon fiber reinforced composites at varying temperatures.

DMTA was used to obtain damping factor (*Tan δ*), complex modulus (E*), tensile compliance (D*), and complex viscosity (η*). The DMTA of carbon fibers across a range of temperatures, from 30 °C to 150 °C, is displayed in Fig. 2.

The magenta curve presents the η* of epoxy reinforced with carbon fibers. It is noted that the complicated viscosity progressively drops as temperature rises until reaching a temperature at which a continues to flow with consistent properties. This effect is characteristic of systems based on polymers, where a rise in heat energy causes molecules to move more freely, reducing viscosity. The viscosity pattern that decreases as temperature rises illustrates how epoxy carbon fibers soften thermally. This is crucial for manufacturing and processing because, at higher temperatures, the material will show less flow resistance, increasing processability.

The damping factor (Tan δ) is represented by the red dotted curve. This value reveals the viscoelastic nature of the carbon fibers by showing the material’s capacity to dissipate energy. Tan δ in the figure increases up to a peak and subsequently falls as temperatures rise. The peak is associated with the glass transition temperature (Tg), which is the temperature at which carbon fibers change from being glassy and stiff to being more flexible and rubbery. Prior T_g_, the epoxy/carbon reinforced fiber becomes softer and loses some of its capacity to release energy. T_g_ is essential for determining the operating temperature range of epoxy/carbon fibers, represented by the peak in the Tan δ curve. The fibers stay stiff below Tg and become flexible above T_g_.

D*, refers to the capacity of material to deform under tensile stress, is displayed on the blue curve. D* rises with temperature, suggesting that epoxy/carbon fibers are more flexible and deformable. This reaction is typical of fiber-reinforced systems and polymers, where higher thermal motion results in increased flexibility. The rising D* shows that as temperature rises, epoxy/carbon fibers become more pliable. In applications where the fibers are subjected to tensile forces, this may lead to decreased mechanical performance at higher temperatures, necessitating careful consideration of operating conditions.

The black curve, which represents the E* of epoxy/carbon fiber reinforced composites, highlights the improved mechanical performance. The epoxy polymer matrix is considerably strengthened by carbon fibers, which raises the E* overall and increases heat stability and damping. Epoxy/carbon fiber-reinforced composites are considered the best choice for satellite applications due to their great strength, longevity, and resistance to deformation under cyclic loading conditions.

**Fig. 3:**
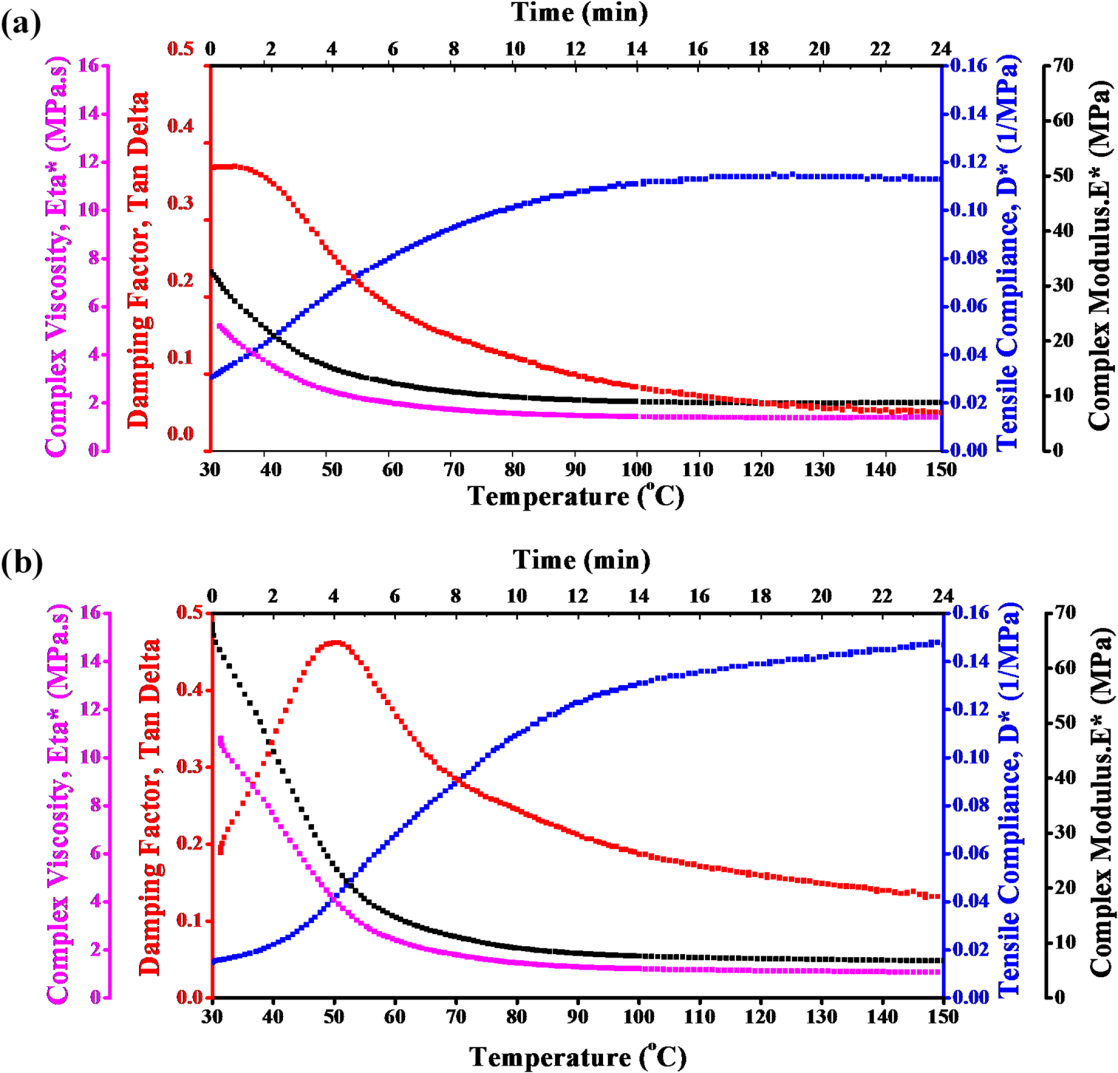
DMTA of epoxy/carbon fiber reinforced composites with (a) 1.5 wt% TiO_2_ and (b) 3 wt% TiO_2_, evaluated across different temperatures.

The DMTA of epoxy/carbon fibers reinforced with 1.5 wt% and 3 wt% TiO_2_ is presented in Fig. 3a and b, respectively. At high temperatures, the decrease of η*, Tan δ, and E* reflect the flexible and less energy dissipated of the epoxy/carbon reinforced fiber with TiO_2_. On the other hand, an increase in D* refers to the reduction in stiffness and increase in flexibility of the polymeric composite reinforced with TiO_2_.

The addition of TiO_2_ to the epoxy/carbon-reinforced fiber influences thermal and mechanical behavior. The DMTA of this reinforced polymeric composite is crucial for understanding how the addition of TiO_2_ influences this behavior. Especially, applications requiring thermal stability and mechanical flexibility, like satellite applications. The data presented can help to optimize the use of epoxy/carbon fiber composites with TiO_2_ additives in high-performance applications where both strength and thermal resistance are critical. The thermomechanical behavior of 1.5 wt% and 3 wt% TiO_2_ is the same. The η* and E* are decreasing with temperature, while the D* has the opposite behavior. The 3 wt% TiO_2_ has higher T_g_ compared to the 1.5 wt%, which reflects the greater mechanical stability at elevated temperatures.

This analysis highlighted the effect of TiO_2_ weight% on the mechanical and thermal behavior of epoxy/carbon-reinforced fiber. Superior stiffness and a higher transition temperature were observed for epoxy/carbon-reinforced fiber with 3 wt% TiO_2_. making it potentially more suitable for satellite applications that require higher thermal stability.

**Fig. 4:**
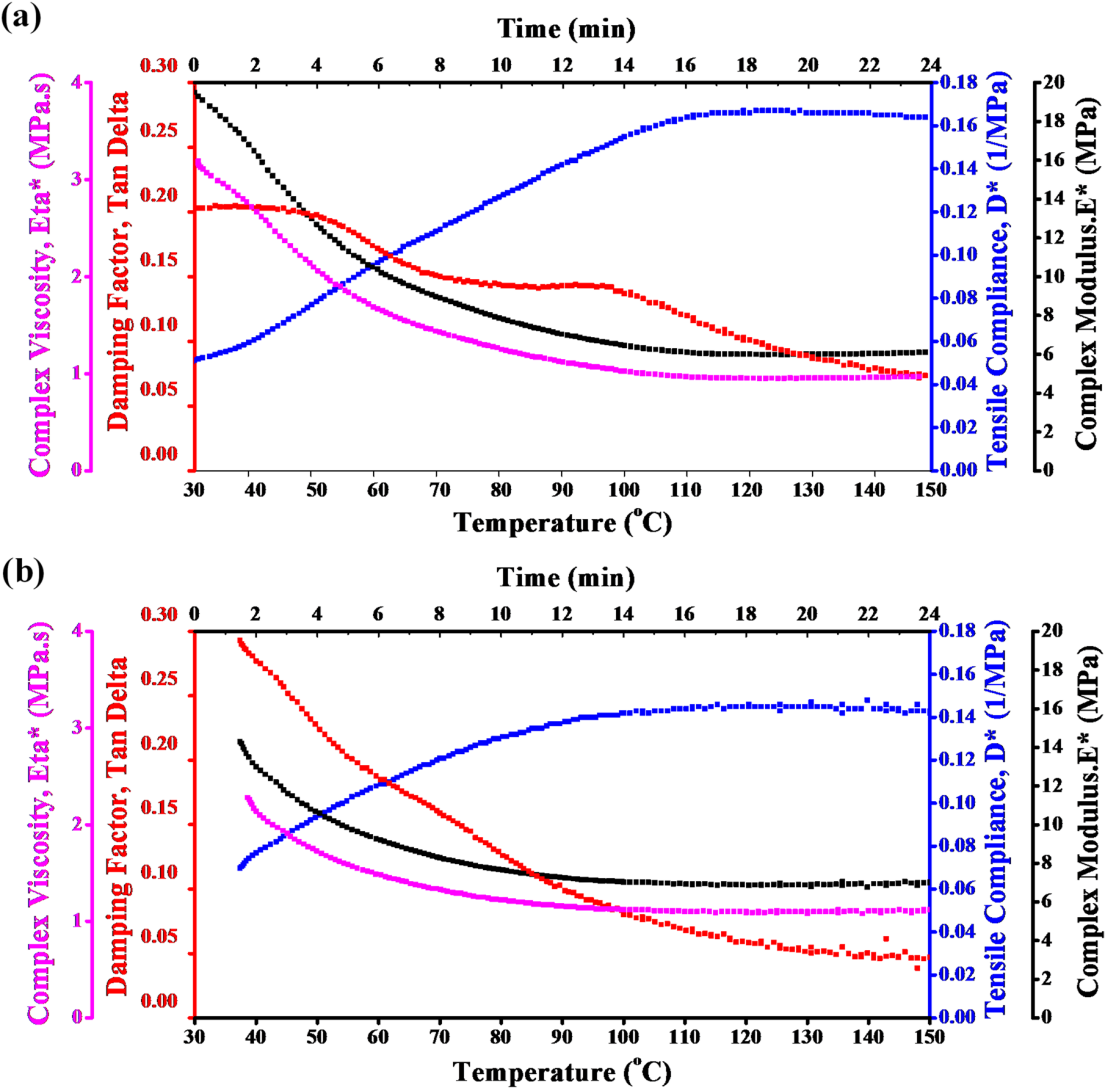
DMTA of carbon fibers at varying temperatures for ZrO_2_ (a) 1.5 wt% and (b) 3 wt%.

The DMTA of epoxy/carbon fibers containing (a) 1.5 wt% ZrO_2_ and (b) 3 wt% ZrO_2_, tested at different temperatures, is shown in Fig. 4. Complex viscosity, which is displayed in purple in both samples, reduces as the temperature rises, as would be predicted given the increased molecular mobility. In comparison to the 1.5G ZrO_2_ sample, the 3G ZrO_2_ sample exhibits a higher initial viscosity, indicating stronger or more strengthened fiber connections.

The glass transition temperature (Tg) is indicated by the damping factor (red) peaks in both samples. When comparing the 3 wt% ZrO_2_ sample to the 1.5 wt% sample, the peak appears at a somewhat higher temperature, indicating that the 3 wt% composite has more thermal stability or rigidity.

In both situations, the tensile compliance (blue) rises with temperature, which is in line with softening behavior. In comparison to the 1.5 wt% ZrO_2_ composite, the 3 wt% ZrO_2_ composite has a lower compliance, suggesting a stiffer material that is more resistant to deformation.

In comparison to the 1.5 wt% ZrO_2_ composite, the 3 wt% ZrO_2_ composite shows improved mechanical properties overall, including lower tensile compliance, a slightly higher Tg (as shown by Tan δ), and a greater complex viscosity. This implies that better stiffness and thermal stability are the outcomes of increasing ZrO_2_ concentration.

**Fig. 5:**
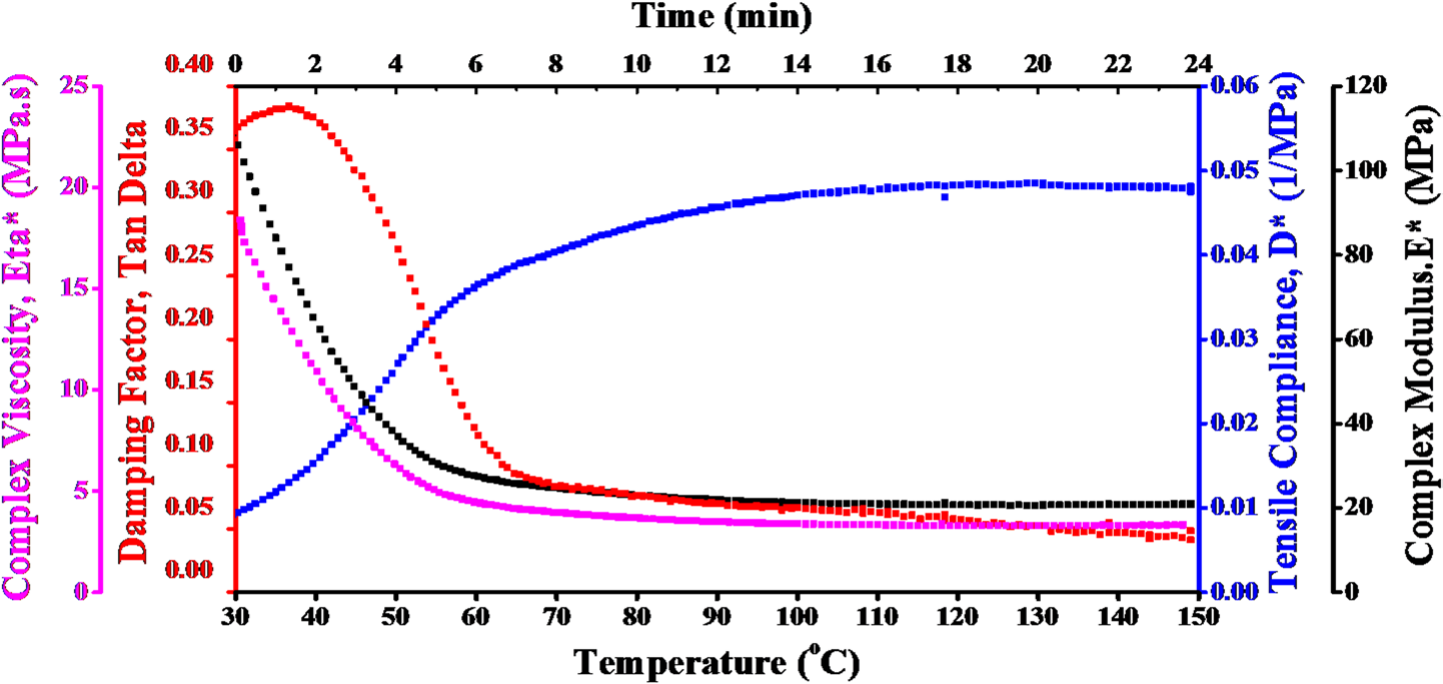
DMTA of carbon fibers containing 3 wt% SiO_2_ at different temperatures.

Figure 5 presents the DMTA of epoxy/carbon fibers containing 3 wt% SiO_2_. The complex viscosity (shown in purple) decreases as temperature increases, indicating a reduction in the material’s resistance to deformation with rising thermal energy. This behavior is typical of viscoelastic materials transitioning from a more solid-like to a more fluid-like state at elevated temperatures.

A peak in the damping factor (red) indicates the glass transition temperature (T_g_) of the material. The peak denotes a transition from a glassy, stiff state to a more springy, flexible one. This T_g_ is necessary to comprehend the material’s thermal stability.

The blue increase in tensile compliance indicates that the material becomes more deformable as the temperature rises. This makes sense since molecules move more freely at higher temperatures, increasing flexibility and decreasing stiffness.

The DMTA results for carbon fibers with 3 wt% SiO_2_ show that complex viscosity reduces, and tensile compliance increases with temperature. The peak in Tan δ indicates the glass transition temperature, which is the temperature at which the mechanical properties of the material change substantially. It is likely that adding 3 wt% SiO_2_ improves the material’s stiffness and thermal stability up to the T_g_.

**Fig. 6:**
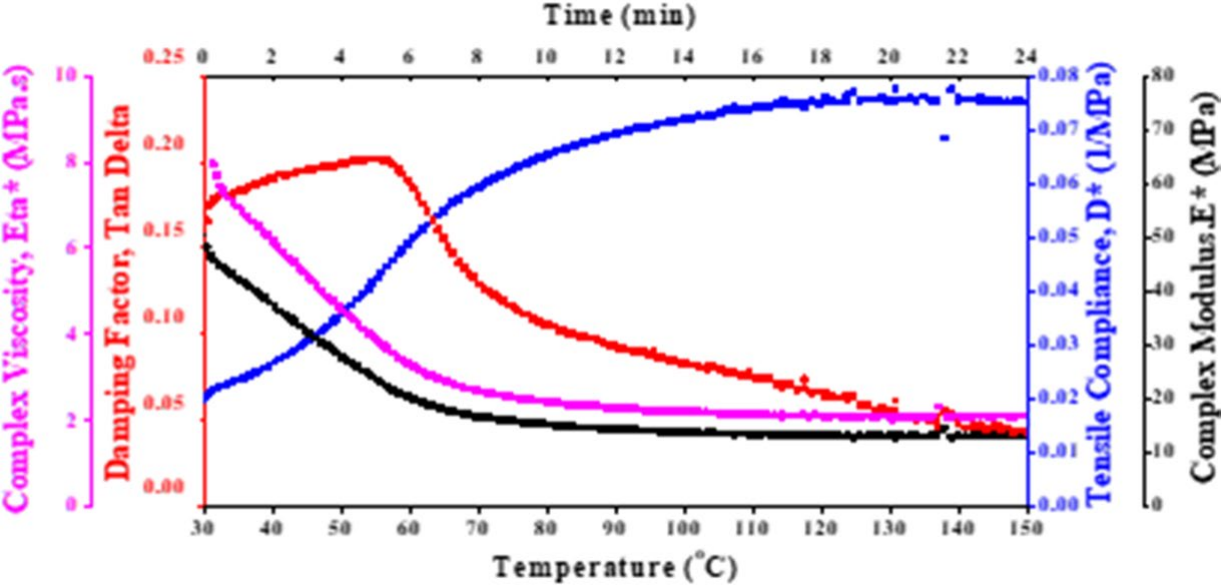
DMTA of carbon fibers containing 1.5wt% graphite at different temperatures.

Figure 6 presents the DMTA of epoxy/carbon fiber reinforced with 1.5 wt% graphite at a temperature range. The DMTA results for carbon fibers containing 1.5 wt% graphite highlights the reinforcing effects of graphite on the composite. At lower temperatures, the graphite improves the composite’s stiffness, as seen in the relatively high complex viscosity and low tensile compliance. Reinforcing the epoxy/carbon matrix with 1.5 wt% graphite increases Tg, Overall, the inclusion of graphite enhances the thermal and mechanical performance of carbon fibers, making them more suitable for applications that demand higher stiffness, reduced compliance, and improved thermal stability.

**Fig. 7:**
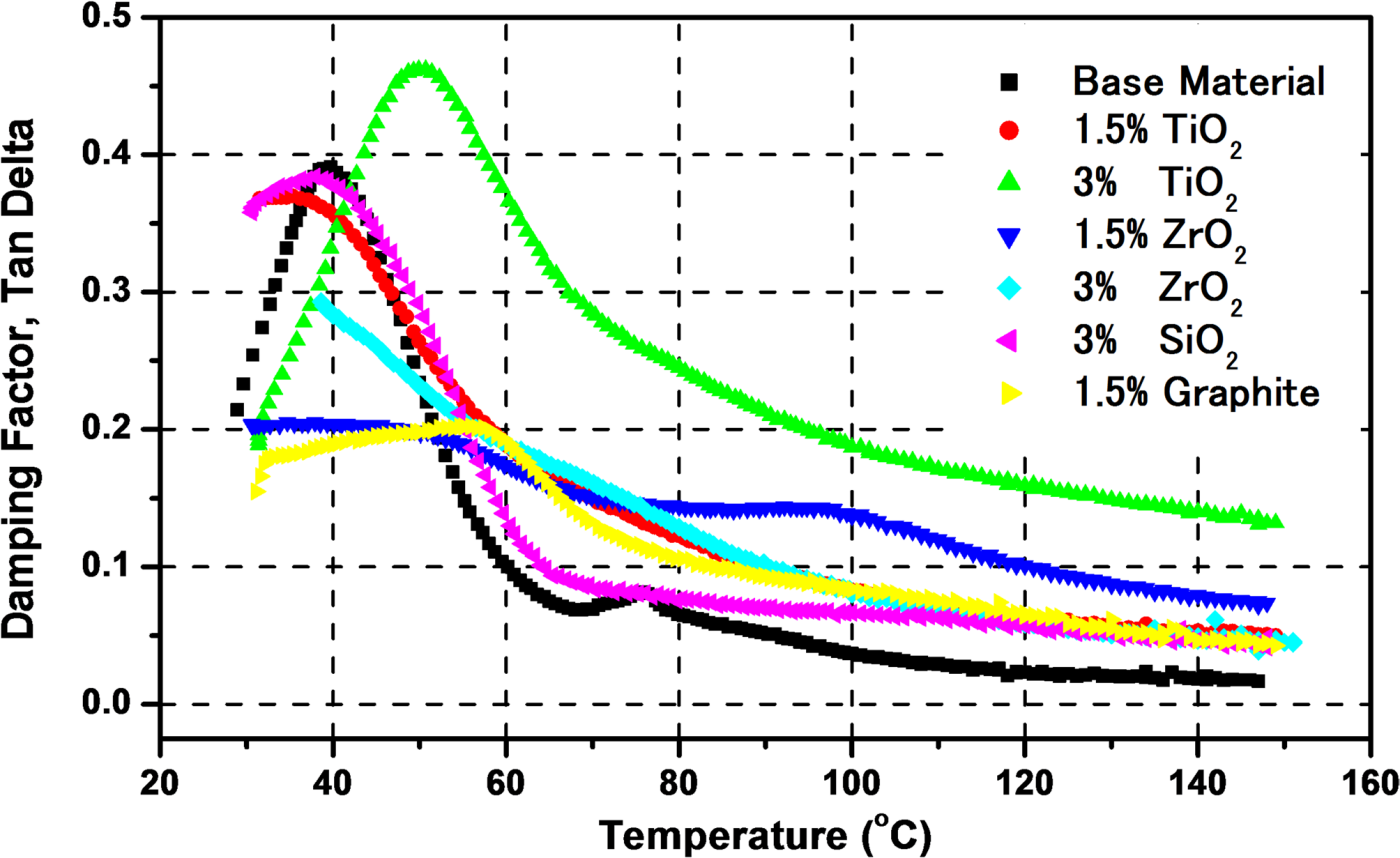
Loss factors for epoxy/carbon reinforced composites.

Figure 7 presents the variation of damping factors with temperature for the processed composite polymers including epoxy/carbon as base material with TiO_2_, ZrO_2_, SiO_2_, and graphite as reinforced materials. Noticeably, the composites exhibit a peak in damping factor at varying temperatures which reflects the transition temperature, where energy dissipation is maximized.

The addition of 1.5 wet. % of TiO_2_ doesn’t have a remarkable effect on the damping factor. However, increasing the concentration to 3 wt% enhanced the damping factor and shifted the transition temperature to higher value which consistent with literature reports demonstrating that nanofillers can restrict polymer chain mobility and enhance stiffness when well-dispersed^22^. The damping factor increased from ~ 0.4 to ~ 0.46 due to this addition and the transition temperature was also changed from ~ 40 °C to ~ 51 °C. In addition, both 1.5 wt% and 3 wt% TiO_2_ had larger damping factor values at temperatures above 50 °C, with the 3 wt% TiO_2_ values being thought to be the highest of all composites. Because of its exceptional qualities, it is advised to utilize it for satellite applications. Moreover, the damping trend of TiO_2_ is valuable for applications where vibration damping is critical, with TiO_2_-reinforced composites being especially effective in such conditions.

ZrO_2_ composites (light blue and cyan lines) have a moderate damping peak. While higher than the base material at temperatures over ~ 53 °C, their damping performance is less pronounced than TiO_2_ composites, indicating a more moderate improvement in energy dissipation. At temperatures below 75 °C, the damping factor for 1.5 wt% ZrO_2_ is higher than that of 3 wt%, and the damping behavior reversed over this temperature.

The 3 wt% SiO_2_ composite (pink line) shows a distinct peak, but it is lower than the 3 wt% TiO_2_ composite. SiO_2_ seems to provide a moderate enhancement in damping but not as significant as TiO_2_ at higher concentrations.

The 1.5 wt% Graphite composite (yellow line) exhibits a comparatively lower peak and a more stable behavior across the temperature range. This implies that although graphite reinforcement enhances damping at higher temperatures, TiO_2_ has a more significant impact.

Generally, an increase in reinforcement concentration (from 1.5 wt% to 3 wt%) results in a higher damping factor. This trend suggests that higher reinforcement levels improve the composite’s ability to dissipate energy, likely due to the enhanced interfacial interaction between the matrix and reinforcement particles. Furthermore, all composites show a decrease in the damping factor at higher temperatures after reaching their peak. This decline might be due to the softening of the matrix, which reduces the material’s ability to absorb vibrational energy effectively.

**Fig. 8:**
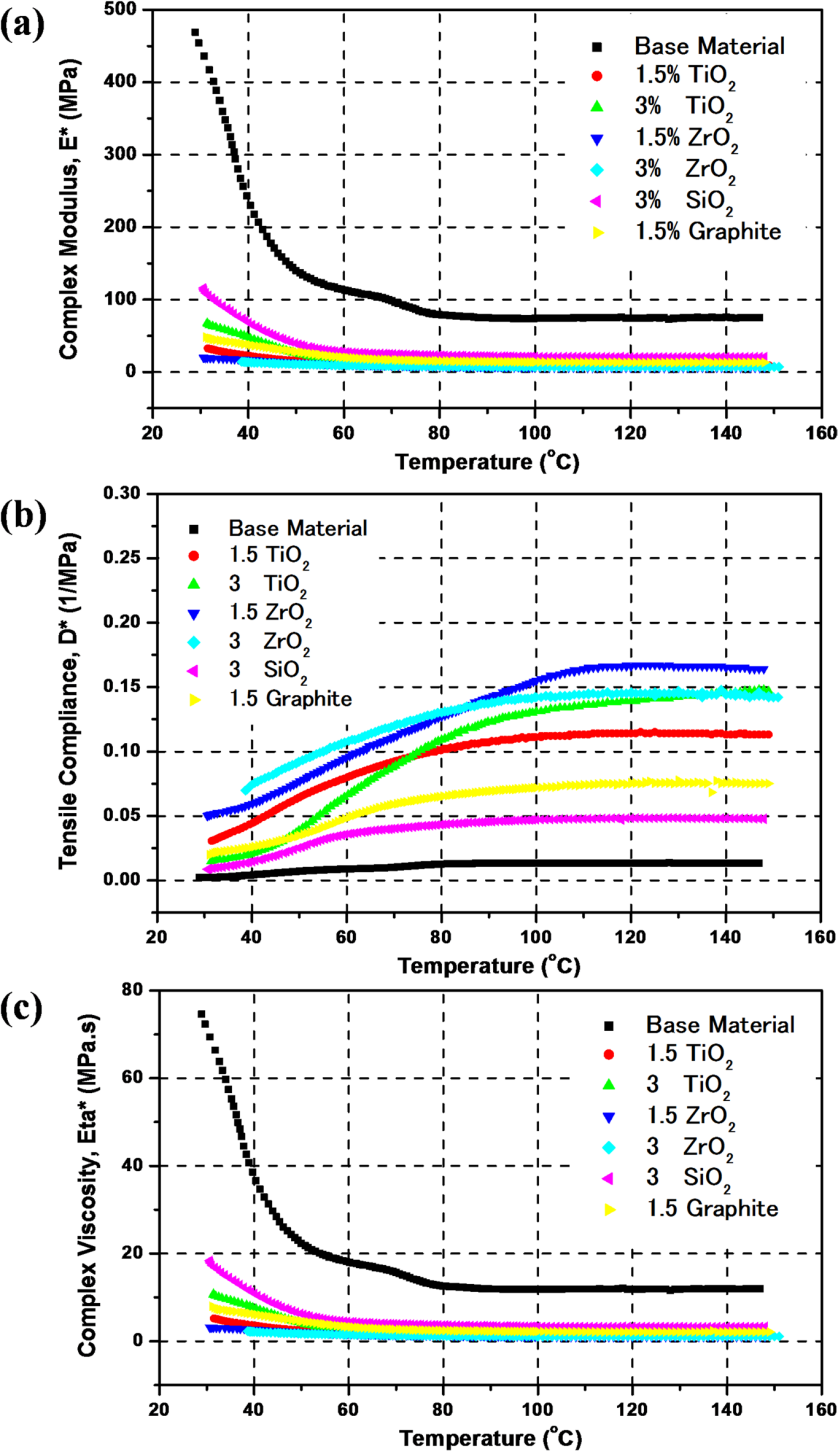
DMTA of epoxy/carbon reinforced composites (a) Complex modulus, (b) Tensile compliance, (c) Complex viscosity.

Figure 8 presents the DMTA of epoxy/carbon and its composites with different concentrations and types of additives, namely TiO_2_ (1.5 wt% and 3 wt.), ZrO_2_ (1.5 wt% and 3 wt%), SiO_2_ (3 wt%), and graphite (1.5 wt%). Three subplots visualize the data: complex modulus, tensile compliance, and complex viscosity.

Figure 8a shows the complex modulus of epoxy/carbon with different nano-additives at a wide temperature range. The complex modulus measures the stiffness of the material under oscillatory loading. Meanwhile Fig. 8b presents the tensile compliance, which is inversely related to stiffness, indicating the material’s ability to deform under stress. So, both figures can work together to provide a clear insight into understanding the DMTA of the processed materials to be used for satellite applications. The base material (epoxy/carbon) has the highest complex modules and the lowest tensile compliance, which reflects its low ability to resist dynamic loading, confirming its higher stiffness and lower deformability. So, the nano-additives were added for this purpose. Generally, the addition of the nano-additives enhanced the epoxy/carbon nano-composite by decreasing the complex modulus and increasing the tensile compliance. At higher temperatures over 80 °C nearly, the tensile compliance improved and increased by the addition of 3 wt% SiO_2_, 1.5 wt% graphite, 1.5 wt% TiO_2_, 3 wt% TiO_2_, 3 wt% ZrO_2_, and 1.5 wt% ZrO_2_, consequently. ZrO_2_ (3 wt%) shows better results compared to TiO_2_ and SiO_2_, highlighting its effectiveness at higher concentrations. Figure 8c illustrates the complex viscosity, which evaluates the material’s resistance to deformation under dynamic conditions. The complex viscosity has the same trend as the complex modulus and the inverse of the tensile compliance.

Table 3 summarizes the key thermomechanical parameters for all the prepared nanocomposites, enabling direct comparison of performance improvements.

**Table 3:** DMTA results for the prepared nanocomposite Epoxy/Carbon fibers.

Composite	Tg (^o^C)	Tan δ	E* (MPa) at 30 °C	D* (MPa) at 150 °C	η* (MPa.s) at 30 °C
Base material	39.7	0.39	469	0.013	74.6
1.5% wt TiO_2_	40	0.40	32.6	0.113	5.19
3% wt TiO_2_	51	0.46	67.9	0.147	10.8
1.5% wt ZrO_2_	34.7	0.21	20	0.164	3.19
3% wt ZrO_2_	38.6	0.29	14.3	0.142	2.28
3% wt SiO_2_	37.9	0.38	11.6	0.048	18.4
1.5% wt Graphite	55.9	0.20	50	0.075	7.95

Table 3 demonstrates that the DMTA responses of the nanofilled composites exhibit clear quantitative trends with respect to nanoparticle type and weight%. The glass transition temperature (T_g_) increases most significantly with graphite and TiO_2_ additions, indicating strong nanoparticle–matrix interactions.

**Fig. 9:**
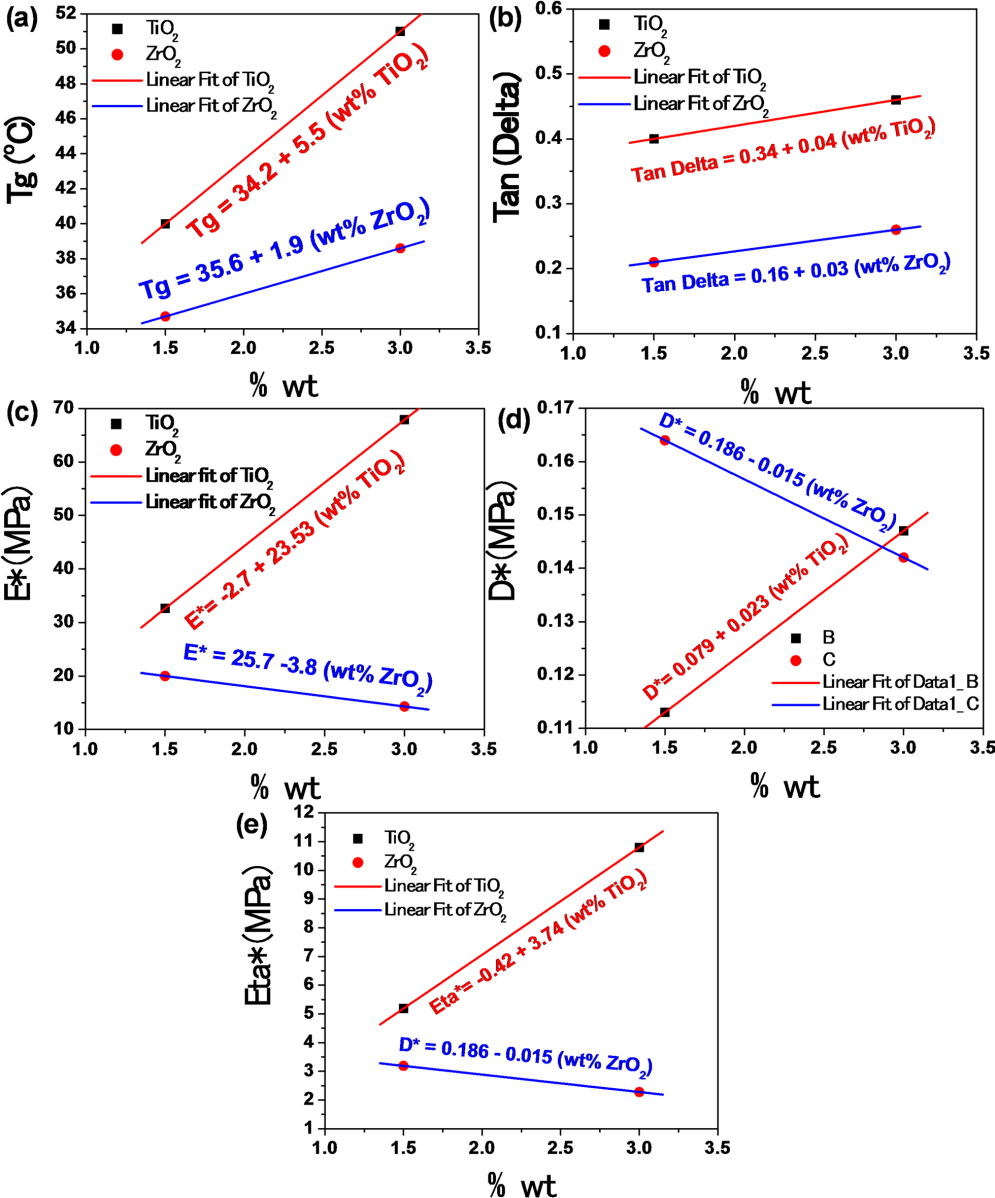
Variation of the viscoelastic parameters of Epoxy/Carbon-Fibers nanocomposites as a function of nanoparticle loading for TiO_2_ and ZrO_2_ fillers obtained from DMTA: (a) T_g_, (b) tan δ, (c) E*, (d) D*, and (e) η*.

To better understand the influence of nanoparticle loading on the thermomechanical behavior of the epoxy nanocomposites, the experimentally measured viscoelastic parameters were fitted using linear relationships, as shown in Fig. 9. The figure presents the experimental data points together with the corresponding fitted curves and their analytical expressions. The incorporation of rigid oxide nanoparticles such as TiO_2_ and ZrO_2_ introduces constraints on the mobility of the polymer chains in the interfacial region surrounding the particles, which leads to modifications in the viscoelastic response of the composite material. As shown in Fig. 9(a), the glass transition temperature (Tg) increases with increasing nanoparticle content, indicating a restriction of segmental motion of the polymer chains due to the presence of the nanoparticles. A similar reinforcing effect is observed for tan δ as indicated in Fig. 9b. E* and η*, which increase with TiO_2_ loading as shown in Fig. 9c-e, reflecting the improved stiffness of the composite due to the load transfer capability of the rigid nanoparticles. In contrast, E* and η* decrease with increasing ZrO_2_. The trend of the D* is inverted if it is compared with the E* curve (D* = 1/E*). The modification of these parameters is associated with interfacial friction and the degree of interaction between the polymer matrix and the nanoparticles. These fitted relationships therefore provide a simplified representation of the experimentally observed trends and facilitate comparison between the different nanoparticle systems, while also highlighting the role of nanoparticle–matrix interactions in governing the viscoelastic behavior of the epoxy nanocomposites.

### Microstructural analysis

#### Optical micrograph

**Fig. 10:**
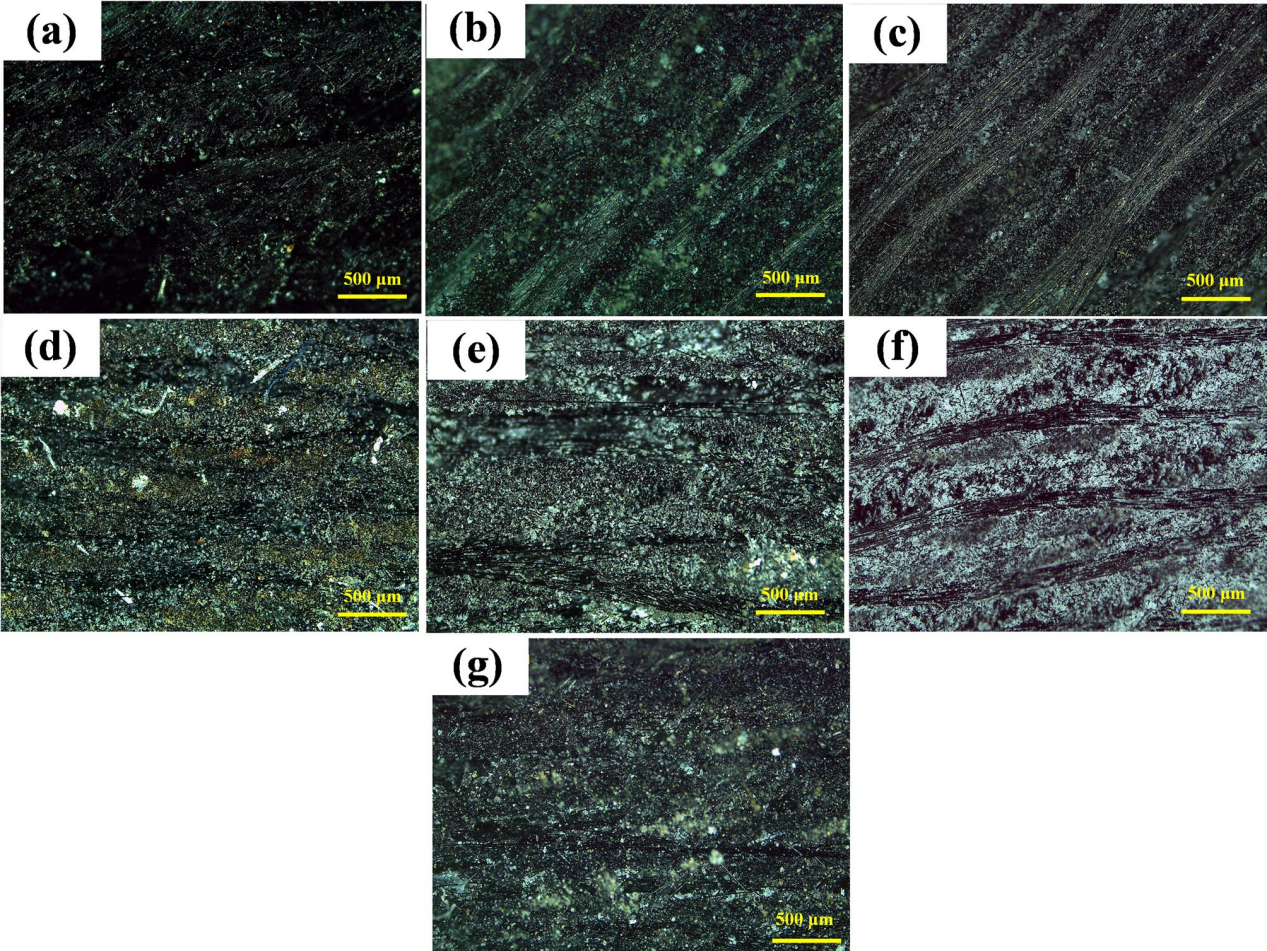
Optical microscopic of the produced polymer matrix composites at: (a) without, (b) 1.5 wt% TiO_2_, (c) 3 wt% TiO_2_, (d) 1.5 wt% ZrO_2_, (e) 3 wt% ZrO_2_, (f) 3 wt% SiO_2_, and (g). 1.5 wt% graphite.

Figure 10 shows the optical micrographs of the fabricated epoxy/carbon fiber without and with the various reinforcement fillers. The current optical micrographs illustrate the microstructural features, dispersion behavior, and potential agglomeration of the reinforcement nanoparticles within the PMC. Despite that the low-magnification optical micrographs are insufficient to conclusively confirm uniform nanoparticle dispersion, as they do not provide adequate resolution to evaluate particle distribution at the micro- and nanoscale levels. So, it will be needed to discover dispersion behavior, and potential agglomeration of the reinforcement nanoparticles within the PMC through SEM.

Figure 10a presents the micrograph of the epoxy/carbon fiber without any nanofiller material. A homogeneous and smooth surface is observed, which reflects the inherent structure of the pure PMC. This micrograph will be considered as a baseline for the PMC with nanofiller.

Figure 10b illustrates the incorporation of 1.5 wt% TiO_2_ nanoparticles into the fabricated epoxy/carbon fibers. Limited agglomeration is observed with well dispersion for the TiO_2_ nanoparticles. The epoxy/carbon fibers show good interfacial compatibility with TiO_2_. Further increase in TiO_2_ content up to 3 wt% is shown in Fig. 10c. The obtained micrograph reveals a higher population of particles with a slightly increased tendency for agglomeration. Although the majority of particles are still evenly distributed, small clusters are noticeable. This clustering could act as stress concentration points under mechanical loading.

Figure 10d depicts the addition of 1.5 wt% ZrO_2_, which are uniformly distributed all over the epoxy/carbon fibers. Such uniform dispersion can contribute positively to enhance the DMTA. Further increases in ZrO_2_ up to 3 wt% is shown in Fig. 10e and provides a visibly denser distribution of particles.

Figure 10f displays the integration of 3 wt% SiO_2_. A relatively fine distribution of the SiO_2_ nanoparticles is observed. However, due to the small size and high surface energy of SiO_2_ nanoparticles, minor clustering is also observed. SiO_2_ can enhance thermal insulation and reduce thermal expansion. The microstructure indicates potential for improved dimensional stability. Figure 10g displays the graphite-reinforced epoxy/carbon PMC with a distinct morphology, with ultrathin, nanoplatelet-like structures visible across the matrix. The dispersion appears uniform, which is crucial for achieving the desired reinforcement effects such as improved DMTA.

#### SEM and EDX characterization

**Fig. 11:**
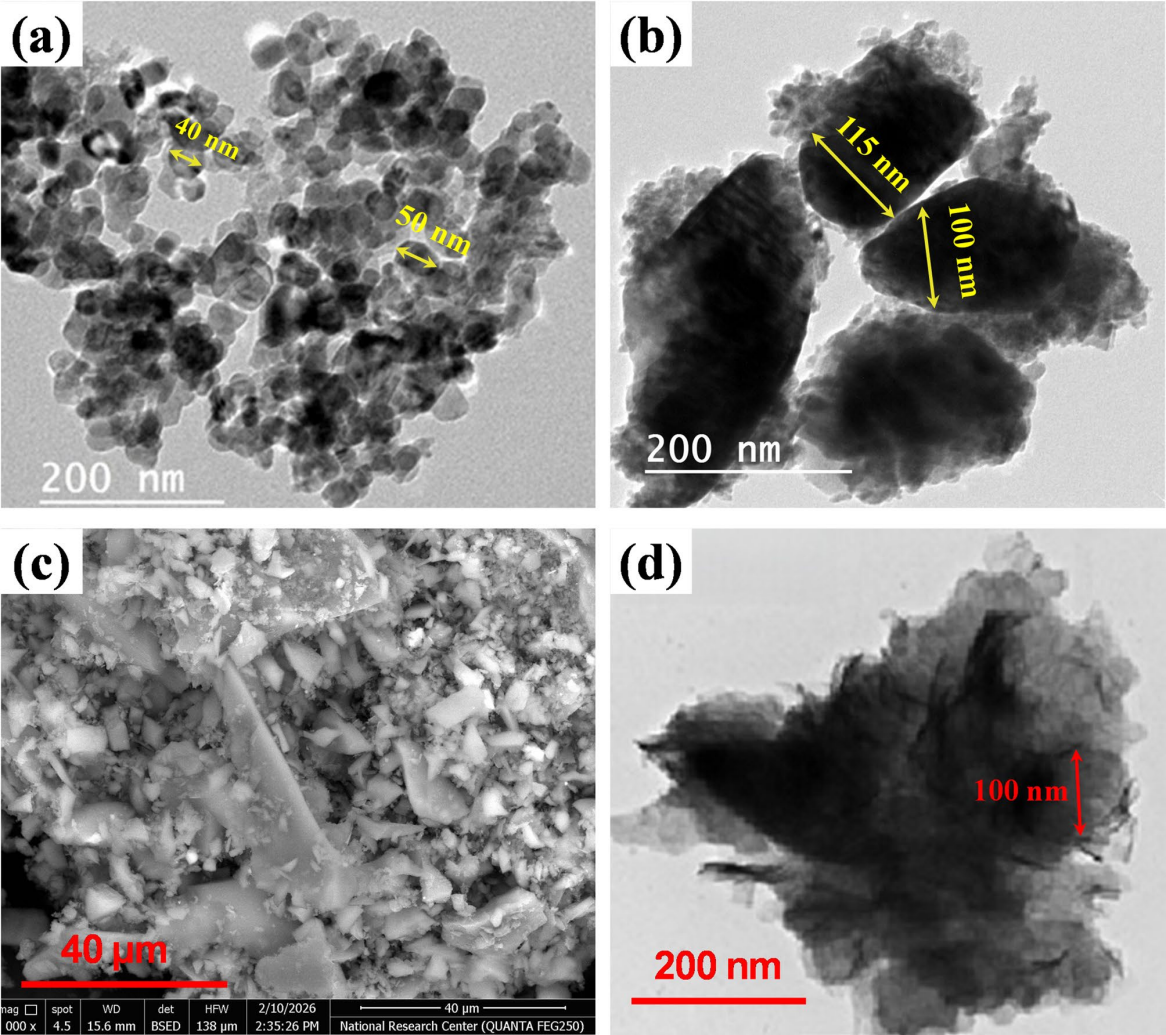
Microscopic images of the nanoparticles used (a) TiO_2_ (b) ZrO_2_ (c) SiO_2_ (d) Graphite.

In the current section, we will examine how the applied nano-additives affect the microstructural characteristics of epoxy/carbon fiber-reinforced polymer. Figure 11 shows SEM images of the nanoparticles used to make the nanocomposites. By measuring the particles in these images, we estimated their sizes and compared them with the values listed in Table 1. Looking at Fig. 11(a), the TiO_2_ nanoparticles are mostly round, with diameters around 40–50 nm. That lines up well with the average size of about 45 nm in Table 1. For ZrO_2_ in Fig. 10(b), the particles look like round clusters, typically measuring 100–115 nm. That’s a bit larger than what Table 1 says for primary particle size, so it looks like some nanoparticles have clumped together. The SiO_2_ particles in Fig. 11(c) are different, they form irregular aggregates that stretch into the micrometer range. Table 1 lists their primary size as nanoscale, so what we’re really seeing are bigger clumps made up of tiny silica particles. On the other hand, the graphite nanoparticles in Fig. 11(d) have the usual flake-like, layered look, with lateral sizes around 100 nm, matching what’s in Table 1. So, the microscopy images back up the idea that these fillers have the nanoscale features described in Table 1, but they also show that some particles tend to stick together more than others.

**Fig. 12:**
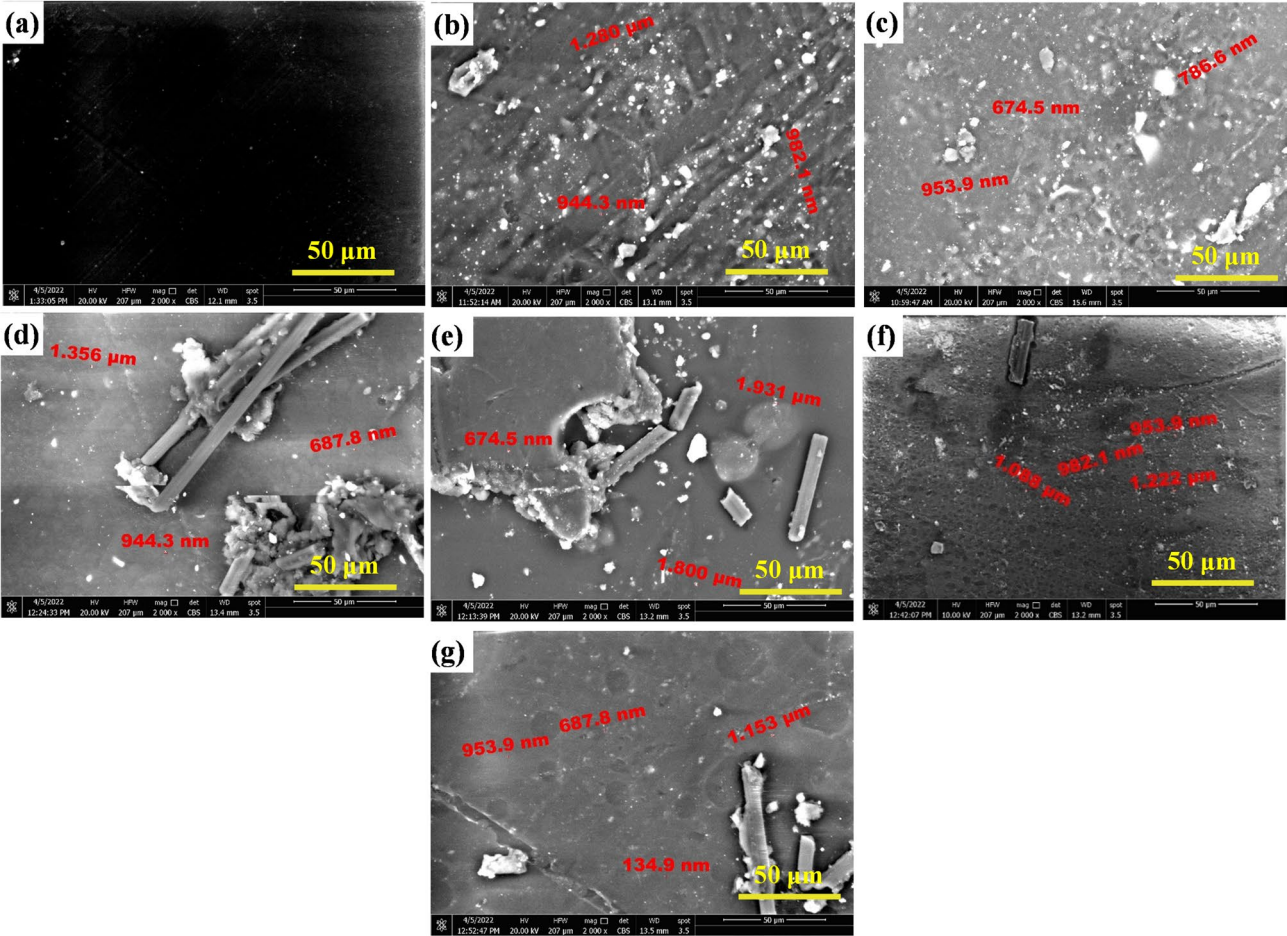
SEM photos of the produced polymer matrix composites with additive at: (a) without, (b) 1.5 wt% TiO_2_, (c) 3 wt% TiO_2_, (d) 1.5 wt% ZrO_2_, (e) 3 wt% ZrO_2_, (f) 3 wt% SiO_2_, and (g) 1.5 wt% graphite.

Figure 12a illustrates the SEM magnified image of the fabricated baseline specimen (epoxy/carbon fiber), without any nanofiller added. There were no voids, defects, porosity, or fiber-matrix debonding observed, which reflects the uniform fiber distribution and effective resin impregnation. The absence of these defects indicates the successful application of the hand lay-up and curing technique. In addition, good fiber wetting and strong interfacial adhesion between the carbon fibers and epoxy matrix are also considered. This good preparation will lead to high mechanical performance, which will be enhanced to a higher extent with the addition of the nano-additives.

Figure 12b and c are the SEM micrographs of epoxy/carbon fibers nanocomposites containing 1.5 wt% and 3 wt% TiO_2_ nanoparticles, respectively. The existence of TiO_2_ nanoparticles is observed to influence significantly the dispersion behavior, particle morphology, and microstructural uniformity of the composite matrix. At the 1.5 wt% TiO_2_ loading, the nanoparticles dispersion is observed to be semi-uniform with local agglomeration in some areas. The sizes of the grains measured range from 944.3 nm to 1280 nm, which are rather big for nano-scale reinforcements as shown in Fig. 12b. In contrast, the microstructure shown in Fig. 12c for the 3 wt% TiO_2_ sample reveals a more homogeneous and acceptable dispersion of nanoparticles in the matrix. The grain sizes therein range from 674.5 nm to 953.9 nm, with a finer and more homogeneous distribution than that of the 1.5 wt% sample. The TiO_2_ nanoparticles are predominantly spherical in morphology, facilitating stable dispersion and enhancing interfacial bonding with the surrounding epoxy matrix. The improved distribution and particle morphology at 3 wt% would allow for more effective stress transfer via the fiber–matrix interface with the potential result of improved DMTA.

The influence of ZrO_2_ nano-additives on microstructural characteristics of epoxy/carbon fibers nanocomposites was evaluated at two concentrations: 1.5 wt% and 3 wt%. Figure 12d and e SEM micrographs provide an idea about the dispersion state and interfacial interaction of ZrO_2_ nanoparticles with carbon fiber and epoxy matrix. For Fig. 12d, the sample with 1.5 wt% ZrO_2_, the microstructure reveals poor dispersion of nanoparticles. There is a strong adhesion of ZrO_2_ particles to carbon fiber surfaces, and severe agglomeration occurs. The agglomerated clusters are irregularly shaped and not well dispersed in the matrix. The presence of such particle agglomerates, particularly if localized at the fiber–matrix interface, can act as stress concentrators with potential to degrade interfacial bonding and reduce load transfer efficiency. Similarly, Fig. 12e presents the microstructure of the 3 wt% ZrO_2_ sample. Despite the higher concentration, the dispersion remains largely unsatisfactory. The ZrO_2_ particles continue to enjoy good interfacial adhesion to carbon fibers and have a propensity to develop larger and more agglomerated structures. Particle dimensions here are as big as 1.931 μm, and elongated rod-like morphologies also occur. Such anisotropic geometries and clustered configurations also detract from the uniformity of the composite and can hinder the DMTA performance via localized stress concentration and compromised matrix continuity.

In general, increasing ZrO_2_ content between 1.5 wt% and 3 wt% did not improve dispersion. Rather, it promoted agglomeration and interfacial incompatibility. This behavior may be either due to ineffective surface modification of the ZrO_2_ nanoparticles or inadequate mixing protocols, both of which are critical to achieve homogeneous dispersion in polymer-based nanocomposites. In order to achieve optimized performance, alternative dispersion techniques or surfactant-assisted mixing may be required to inhibit clustering and enhance nanoparticle-matrix interaction.

The impact of addition of 3 wt% SiO_2_ nano-additives on epoxy/carbon fibers nanocomposites was investigated to examine their dispersion behavior and impact on microstructural characteristics. Figure 12f is the SEM micrograph of composite containing 3 wt% SiO_2_ nanoparticles. Dispersion quality of SiO_2_ at this weight% was fairly good. While the particles were more or less evenly dispersed in the epoxy matrix, there were more densely packed local areas seen, indicating some degree of aggregation. The distribution, however, was still more even than that seen in samples with ZrO_2_ nano-additives.

The particle sizes of these SiO_2_ nanoparticles ranged from approximately 953.9 nm to 1222 nm, which is within but quite high for nano-scale additions. This suggests that while particles may have undergone partial agglomeration during mixing or curing, the formation of highly large-sized clusters harmful to mechanical properties was not apparent. Morphologically, SiO_2_ nanoparticles were predominantly in semi-spherical shape, which is preferable to minimize points of stress concentration and ensure greater interaction with the surrounding epoxy matrix. This geometry will provide relatively stable embedment into the resin and may minimize points of microcrack appearance during mechanical loading.

The microstructural analysis of the epoxy/carbon fibers nanocomposite reinforced with 1.5 wt% graphite, as shown in Fig. 12g, shows the formation of clear agglomerated regions. The agglomerates consist of graphite exhibiting dendritic and whisker-like morphologies, which confirm uneven dispersion within the epoxy matrix. Irregular shapes show that the nanoparticles can be clustered due to inadequate dispersion in the matrix. In addition to these features, round regions with entrapped air bubbles also occur. These features are the result of unsatisfactory interface compatibility or poor wetting between the graphite and matrix. Entrapped air bubbles are most likely due to density and surface energy differences between the graphite and epoxy resin, leading to a failure to evacuate voids in hand lay-up and curing.

Quantitative image analysis revealed strong correlations between nanoparticle dispersion metrics and mechanical response. Improved dispersion increased E*, reduced high-temperature damping, and produced more stable viscosity behavior, demonstrating the critical role of nanoparticle distribution in controlling composite performance.

**Fig. 13:**
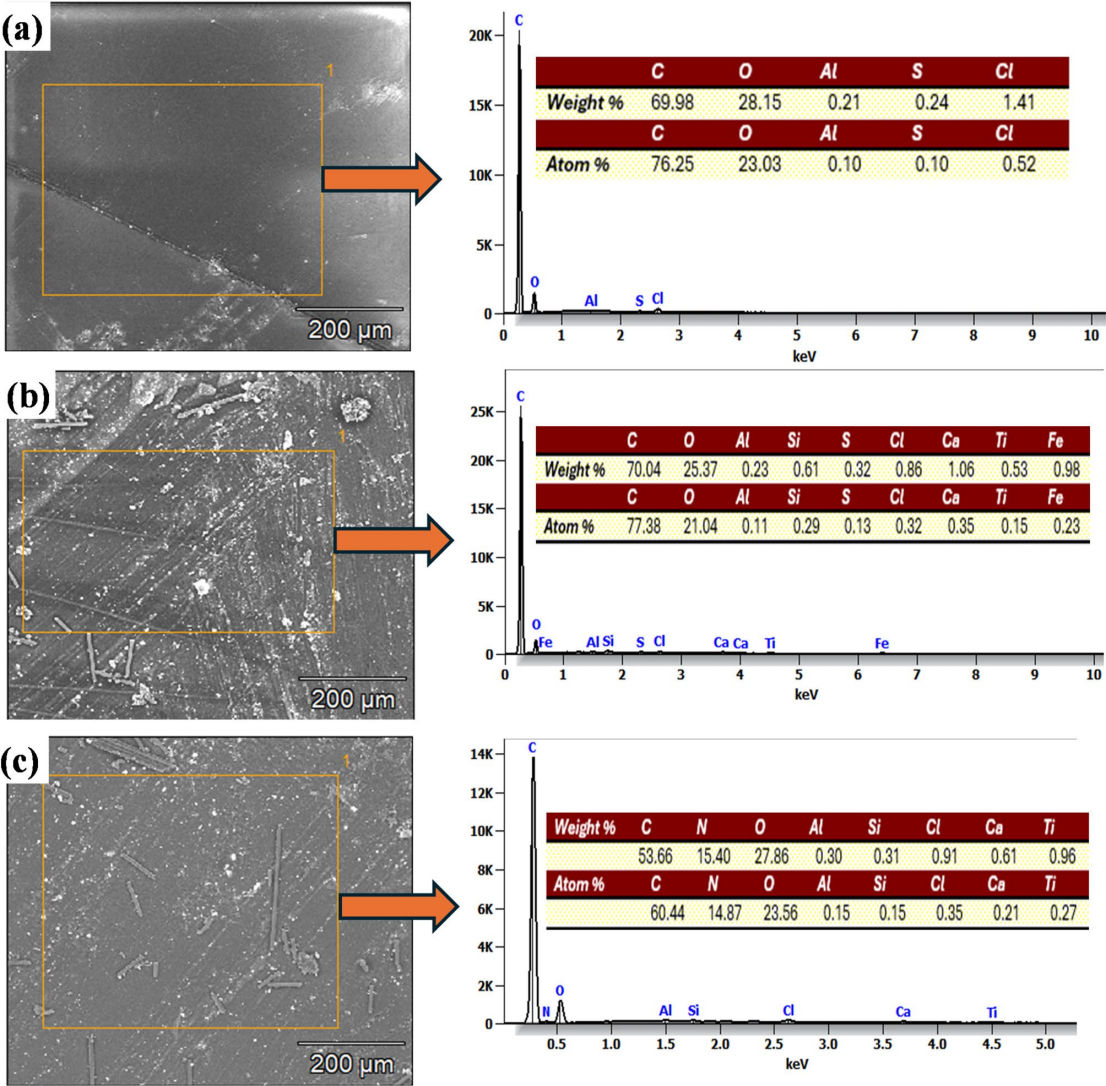
EDS elemental mapping analysis of the produced polymer matrix composites with additive at: (a) without, (b) 1.5 wt% TiO_2_, (c) 3 wt% TiO_2_, (d) 1.5 wt% ZrO_2_, (e) 3 wt% ZrO_2_, (f) 3 wt% SiO_2_, and (g) 1.5 wt% graphite.

Elemental mapping was performed using Energy-Dispersive X-ray Spectroscopy (EDS) integrated within the SEM system to visualize the spatial distribution of the nanoparticles within the composite. Unlike point analysis, which provides elemental information at a single location, EDS mapping scans the entire micrograph area and generates color-coded distribution maps for selected elements (e.g., Ti, Zr, Si, or C). These maps allow direct observation of nanoparticle dispersion, identification of agglomerated regions, and verification of particle–matrix interactions across the fractured surface. In this study, EDS maps were used specifically to confirm the presence and uniformity of the added nanofillers and to distinguish nanoparticle-rich zones from the surrounding epoxy and carbon-fiber regions. Thus, in the sample without nano-additives, the elements of C, O, Al, S, and Cl were unsatisfactory distribution in the whole surface of prepared sample as shown in Fig. 13a, this is caused to the appearance of voids as shown in Fig. 13a. For instance, in the sample of 1.5 wt% TiO_2_nano-additives, the distribution of elements, including TiO_2_, was uniform in the whole surface of the prepared sample, as shown in Fig. 13b. In addition, the increasing of the wt% of TiO_2_ to 3% was also confirmed as illustrated in Fig. 13c. This improvement is attributed to good bonding between TiO_2_ nano additives with carbon fiber and epoxy.

#### Fractographic analysis

**Fig. 14:**
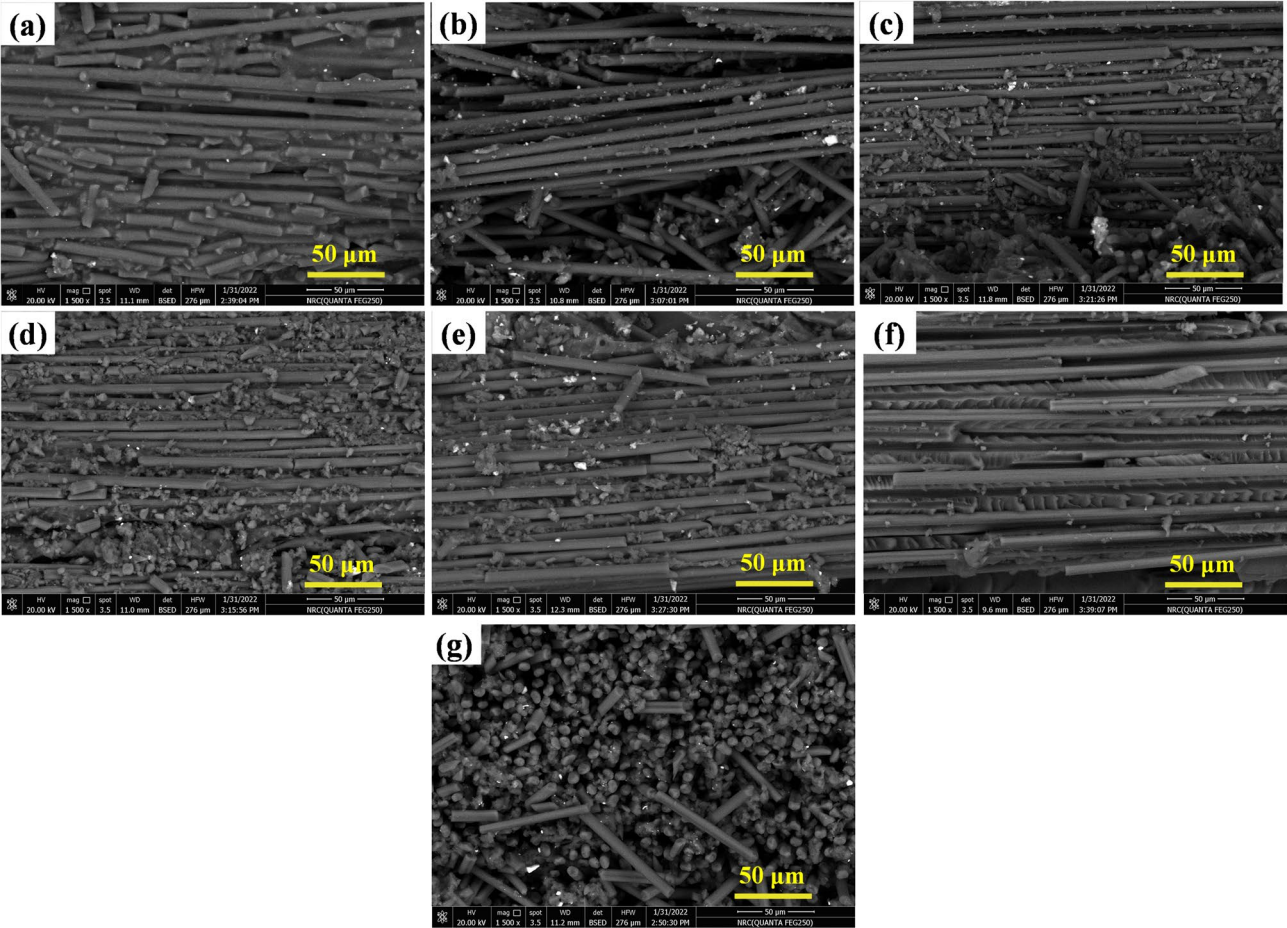
SEM Fractography of the produced polymer matrix composites at: (a) without, (b) 1.5 wt% TiO_2_, (c) 3 wt% TiO_2_, (d)1.5 wt% ZrO_2_, (e) 3 wt% ZrO_2_, (f) 3 wt% SiO_2_, and (g) 1.5 wt% graphite.

The fracture surfaces of the nano-reinforced composites were examined using SEM to analyze failure mechanisms and nanoparticle–matrix interactions as shown in Fig. 14. The fracture analysis provides clear insight into the fracture mechanisms, nanofiller dispersion, and interfacial adhesion between the matrix, reinforcing fibers, and the added nanoparticles.

Figure 14a presents the fracture surface on the Epoxy/Carbon fiber composite without any nano-additives to work as a baseline for the other matrices with nono-composites. The surface exhibits typical features of fiber-reinforced polymers, including some degree of fiber pull-out and exposed matrix regions, indicating the inherent fiber-matrix interfacial strength and the composite’s response to fracture without the influence of additional nanoparticles.

Figure 14b and c present the incorporation of 1.5 wt% and 3 wt% of TiO_2_ nanoparticles into the Epoxy/carbon fiber composite. The presence of the TiO_2_ nanoparticles is attributed by the bright particles dispersed within the polymer matrix and occasionally adhering to the fiber surfaces. The dispersion of the TiO_2_ nanoparticles within the Epoxy/carbon fiber is good. The TiO_2_ nanoparticles are working as obstacles or crack deflection sites which will increase the roughness of the fracture surface compared with the base line sample. The higher concentration of the TiO_2_ nanoparticles might lead to increased particle-matrix or particle-fiber interactions. The effectiveness of this higher loading depends on maintaining good dispersion; any significant agglomeration could potentially act as stress concentrators, thereby altering the composite’s mechanical response.

Figure 14d presents the fracture of samples containing 1.5 wt% ZrO_2_ nanoparticles which are distributed throughout the matrix. These nanoparticles have a great potential to enhance fracture toughness by promoting crack pinning or deflection mechanisms. A greater number of ZrO_2_ nanoparticles (3 wt%) are visible on the fracture surface as shown in Fig. 14e. The increased concentration of these particles could lead to a more pronounced effect on the composite’s fracture behavior. Maintaining uniform dispersion at this higher loading is crucial to avoid detrimental effects from particle clustering.

The fractography of the composite with 3 wt% SiO_2_ nanoparticles (Fig. 14f) reveals a relatively uniform dispersion of these particles within the polymer matrix. SiO_2_ nanoparticles are commonly employed to improve the stiffness, hardness, and thermal stability of composites. The observed dispersion suggests a favorable interaction that could contribute to enhanced load transfer and crack resistance.

Figure 14g presents a distinctly different fracture morphology compared to the other samples. The surface is characterized by a very high density of small, irregularly shaped features, consistent with the presence of 1.5 wt% graphite. This suggests that graphite, even at a relatively low concentration, significantly alters the fracture path. The unique two-dimensional structure and high aspect ratio of graphite can lead to complex interactions within the matrix, potentially promoting crack bridging, crack branching, or increased energy dissipation during fracture. The apparent higher density and distinct morphology of the graphite structures indicate a strong influence on the composite’s failure mechanism, possibly due to the formation of a more intricate network or localized reinforcement.

## Conclusion

This study demonstrated the incorporation of different nanoparticle types (TiO_2_, ZrO_2_, SiO_2_, and graphite) into carbon-fiber/epoxy laminates to investigate the thermomechanical responses. The results show that nanoparticle reinforcement does not follow a universal trend; rather, each filler influences the T_g_, Tan δ, E*, D* and η* in a unique manner.

Among all fillers, 1.5 wt% graphite produced the highest improvement in thermal resistance, increasing Tg from 39.7 °C to 55.9 °C, confirming its strong ability to restrict polymer chain motion. TiO_2_ was the most effective oxide filler for enhancing both Tg and storage modulus, with 3 wt% TiO_2_ increasing Tg to 51 °C and raising E* from 32.6 MPa to 67.9 MPa relative to the 1.5 wt% loading. In contrast, ZrO_2_ primarily improved high-temperature damping, with 1.5 wt% ZrO_2_ producing the highest D* (0.164 MPa) at 150 °C, indicating enhanced viscous dissipation at elevated temperatures. SiO_2_ maintained a balanced response with moderate Tg (37.9 °C) and the highest complex viscosity (18.4 MPa·s), consistent with its strong interphase and surface-energy-driven interactions with the epoxy matrix.

## Supplementary Information

Below is the link to the electronic supplementary material.

Supplementary Material 1

## Data Availability

The datasets generated and/or analysed during the current study are available in the [Supplementary material]”.
